# Real-world effectiveness of intravenous belimumab in adults with systemic lupus erythematosus: results of the observational OBSErve study in the Russian Federation

**DOI:** 10.1186/s41927-024-00452-0

**Published:** 2025-01-08

**Authors:** Alexander Mikhailovich Lila, Elena Aleksandrovna Aseeva, Alyona Igorevna Zagrebneva, Irina Borisovna Vinogradova, Ruzana Ramilovna Samigullina, Munther Khamashta, Tamer Elfishawy, Lindsey Teichman, Debora dos Santos, Juliana Queiroz, Larisa Alexandrovna Kniazeva, Saeed Noibi

**Affiliations:** 1 Institute of Rheumatology by V.A. Nasonova, Russian Academy of Science, Moscow, Russian Federation; 2 Moscow City Clinical Hospital №52, Ministry of Health of Moscow, Moscow, Russian Federation; 3 Ulyanovsk Regional Hospital, Ulyanovsk, Russian Federation; 4 I.I. Mechnikov Northwestern State Medical University, St. Petersburg, Russian Federation; 5 GSK, Medical Affairs, Dubai, United Arab Emirates; 6 GSK, Real World Matrix Team, Arenco Tower, 19th Floor, Sheikh Zayed Road, PO Box 50199, Dubai, United Arab Emirates; 7 GSK, Real World Matrix Team, Rio de Janeiro, Brazil; 8 GSK, Emerging Markets, Moscow, Russian Federation; 9 GSK Saudi Arabia, Value Evidence and Outcomes, Jeddah, Saudi Arabia

**Keywords:** Belimumab, Biologic drugs, Cohort study, Disease activity, Disease modification, Glucocorticoids, Healthcare resource utilization, Lupus flare, Real-world data, Retrospective study

## Abstract

**Background:**

The real-world effectiveness of intravenous (IV) belimumab in treating systemic lupus erythematosus (SLE) has been demonstrated in various countries through the OBSErve (evaluation Of use of Belimumab in clinical practice SEttings) program. Here we describe the clinical effectiveness of IV belimumab for treating SLE in real-world clinical practice in the Russian Federation.

**Methods:**

In the retrospective, observational OBSErve Russia study (GSK Study 215349), eligible physicians enrolled adults with SLE receiving IV belimumab as part of their standard care. De-identified data were collected from patient medical records from September 2021 to March 2022. The primary outcome was the physician-assessed overall clinical response at 6 months post-index versus index (belimumab initiation) among patients receiving belimumab for ≥6 months. Other endpoints included change in Safety of Estrogens in Lupus Erythematosus National Assessment – SLE Disease Activity Index (SELENA-SLEDAI) score and glucocorticoid use.

**Results:**

Overall, 59 patients initiated IV belimumab, mainly due to the previous regimen not being effective and to decrease glucocorticoid use (76.3% each); 15.3% of patients started belimumab within the first year of SLE diagnosis. Only 13.6% of patients discontinued belimumab within the first 6 months, mainly due to loss to follow-up and loss of insurance/reimbursement. At 6 months post-index, among patients who completed ≥6 months of belimumab therapy (full analysis set, *n* = 53), 90.6% and 60.4% had an overall clinical improvement of ≥20% and ≥50%, respectively. Mean (standard deviation, SD) change in SELENA-SLEDAI score from index to 6 months post-index was −5.9 (4.3). Mean (SD) glucocorticoid dose decreased from 12.2 (7.3) mg/day at index to 8.6 (5.1) mg/day at 6 months post-index (*n* = 50).

**Conclusions:**

Patients with SLE receiving IV belimumab for 6 months in real-world settings in the Russian Federation experienced overall clinical improvements and reductions in glucocorticoid use, which is an important long-term strategy of SLE treatment.

**Supplementary information:**

The online version contains supplementary material available at 10.1186/s41927-024-00452-0.

## Background

Systemic lupus erythematosus (SLE) is an autoimmune, chronic, inflammatory disease that affects multiple organs and tissues [1]. Standard therapy, such as glucocorticoids, antimalarials, and other immunosuppressive medications, focuses on managing symptoms, reducing disease activity, preventing organ damage, and improving patient health-related quality of life [2]. Despite these treatment options, some patients with SLE continue to accrue irreversible organ damage over time [3]. Furthermore, as prolonged use of glucocorticoids has been shown to lead to further damage, a major treatment goal for SLE is to reduce the use of glucocorticoids when clinically possible [4]. With these conventional symptom-targeting treatments carrying an increased risk of irreversible damage to the patient, there remains an unmet need for additional, disease-modifying therapies that can target the underlying cause of SLE and, in doing so, reduce disease activity, prevent organ damage, and improve long-term outcomes.

Belimumab, a human recombinant immunoglobulin G1λ (IgG1λ) monoclonal antibody that inhibits soluble B-lymphocyte stimulator (BLyS), is approved as an add-on therapy in over 75 countries for adult patients with SLE [5]. It is also approved for patients ≥5 years of age with SLE in several countries and for adults and children ≥5 years of age with active lupus nephritis (LN) [6]. Intravenous (IV) belimumab has consistently demonstrated greater efficacy compared with standard therapy alone across a number of Phase 3, randomized, placebo-controlled clinical trials [7–9]. In a real-world setting, the effectiveness of IV belimumab in the treatment of SLE was demonstrated through the OBSErve (evaluation Of use of Belimumab in clinical practice SEttings) program that comprised separate observational studies in various countries with broadly similar protocols but with individual, country-specific designs and operational considerations. These individual studies all showed high physician-reported response rates, lower disease activity and glucocorticoid use, and improvement in various organ manifestations of SLE following 6–24 months of belimumab treatment [10–15].

In the Russian Federation, IV belimumab has been in clinical use for the treatment of SLE since 2015 [5, 16]; however, IV belimumab effectiveness and utilization data from real-world clinical practice settings are limited. Therefore, similarly to the other real-world setting OBSErve studies, this study aimed to describe the impact of IV belimumab on clinical outcomes following 6 months of treatment as part of standard therapy in patients with active SLE within the context of everyday healthcare practices in the Russian Federation.

## Methods

### Study design

**Fig. 1:**
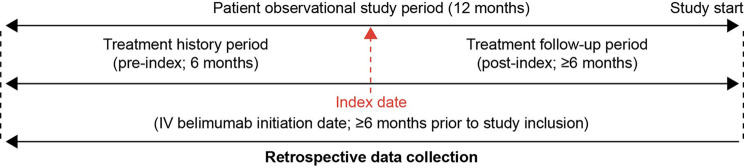
OBSErve Russia study design. *IV* intravenous

OBSErve Russia was a multicenter, observational study (GSK Study 215349) that was designed to retrospectively collect real-world information from patient medical records on the short-term outcomes of IV belimumab use in patients with active SLE (Fig. 1). De-identified data were collected from historical patient medical records and entered into electronic case report forms (eCRFs) by the study physician from September 2021 to March 2022. The study period consisted of a 12-month observational period that comprised 6 months before index (index = date of IV belimumab initiation) (treatment history period) and 6 months after index (follow-up period, i.e., reflection of how long ago the patient started on belimumab treatment and how long they remained on it). The collected data included patient demographics, clinical characteristics (including clinical assessment of SLE severity and activity based on physician’s judgement), comorbidities, clinical and immunologic SLE manifestations, IV belimumab treatment pattern, treatment outcomes, medication use, and healthcare resource utilization (HCRU).

This study was conducted in accordance with the Guidelines for Good Pharmacoepidemiology Practices and the Declaration of Helsinki 2008 and approved by the ethical committees and/or institutional review boards in line with local requirements. Informed consent was not required as the study was non-interventional with data de-identified; the ethics committee of each principal investigator was consulted to confirm this decision.

### Objectives

The primary objective was to evaluate the physician-assessed overall clinical response to IV belimumab at 6 months post-index among patients receiving IV belimumab for ≥6 months, where clinical response was assessed using a physician-deemed clinical improvement scale, similar to a visual analog scale, and categorized as worse, no improvement, <20% improvement (minimally improved), 20–49% improvement (clear but moderate improvement), 50–79% improvement (great improvement), or ≥80% (nearly normalized).

The secondary objectives were to describe characteristics of IV belimumab treatment (reasons for initiation and discontinuation); disease activity assessment (Safety of Estrogens in Lupus Erythematosus National Assessment—SLE Disease Activity Index [SELENA-SLEDAI]; Physician Global Assessment [PhGA] Scale; Systemic Lupus International Collaborating Clinics/American College of Rheumatology [SLICC/ACR] Damage Index [SDI]) at index and 6 months post-index; number of tender and swollen joints 6 months pre- and post-index; number and severity of SLE flares (defined based on the SELENA-SLEDAI flare composite [17], definition provided in Additional file 1) 6 months pre- and post-index; treatment patterns of concomitant medications, particularly glucocorticoid use and dose change, at and/or prior to index and during 6 months post-index; change from index to 6 months post-index in laboratory results (anti-dsDNA, complement C3/C4); and HCRU (the number of scheduled and unscheduled outpatient visits, hospitalizations, and emergency room visits) 6 months pre- and post-index.

### Study physicians

Physicians were invited to participate in this study if they were currently managing ≥10 patients with SLE, had ≥5 years of experience treating SLE, had treated ≥2 patients with IV belimumab as part of standard care, and were treating ≥1 patient with IV belimumab as standard care at the time of recruitment. Eligible physicians also had to agree to all requirements of the study, including resolution of data validation queries.

### Study patients

Patients were consecutively enrolled starting with the most recent patient to have completed 6 months of IV belimumab treatment, until each site’s enrolment target was reached. Physicians enrolled all patients from their practices who fulfilled the following criteria: ≥18 years of age; confirmed SLE diagnosis; previously belimumab-naïve; received their first IV belimumab administration as part of standard care ≥6 months before inclusion into the study; had received ≥1 dose of IV belimumab; and had documented medical records for the duration of the study follow-up period. No specific classification criteria/instruments for SLE diagnosis were required as the physician at enrolment may have differed to the diagnosing physician, and thus, the criteria used for diagnosis may not have been available in medical records.

Patients who received subcutaneous (SC) belimumab at any point during the study, who enrolled in an SLE-related clinical trial during the 12-month patient observational period, or who started IV belimumab as part of a clinical trial, were excluded from this study. Patients were also excluded if they had severe active LN (defined as: proteinuria >6 g/24 h or equivalent using spot urinary protein-creatinine ratio or serum creatinine >2.5 mg/dl; or active nephritis; or requirement for hemodialysis or prednisolone dose >100 mg/day) or kidney disease within 90 days before the index date, or a documented record of severe central nervous system lupus (including seizures, psychosis, organic brain syndrome, and cerebrovascular accident) within 60 days before the index date.

All patients who met the inclusion criteria were considered for inclusion to minimize patient selection bias. Sites consecutively enrolled patients starting with the most recent patient to have completed 6 months of IV belimumab treatment and worked backwards until the site’s target was reached to ensure that the most complete medical history was available.

### Statistical analysis

The target sample size was 50–80 patient medical records for the extraction of anonymized data. Patient enrolment stopped once the approximate target number of patients was achieved. No formal sample size estimation was conducted owing to the descriptive nature of the study.

Descriptive statistics such as counts and percentages and 95% confidence intervals (CI) were used to analyze categorical data, and mean, standard deviation (SD), and median and minimum and maximum were used to analyze continuous data. No imputation of missing data was used.

Patients who completed ≥6 months of IV belimumab treatment were included in the full analysis set (FAS; used to support primary and some secondary endpoints). Patients who discontinued IV belimumab prior to 6 months post-index were included in the enrolled set (used in the analyses of baseline patient demographics and clinical characteristics, IV belimumab treatment pattern, and concomitant medication use).

## Results

### Patient population

Four physicians across four sites enrolled 59 eligible adult patients with SLE (enrolled set), of whom 53 completed ≥6 months of IV belimumab treatment and were included in the FAS.

In the enrolled set, the majority of patients were female (89.8%) and mean (SD) age was 36.8 (10.2) years. Most patients had persistent SLE disease activity (66.1%) and moderate disease severity (64.4%) at IV belimumab initiation (Table 1). Of the 59 patients enrolled, 71.2% had ≥1 comorbid condition pre-index, with Sjogren’s syndrome (28.6%), antiphospholipid syndrome (26.2%), and hypertension (21.4%) being the most frequently reported among patients with comorbid condition (*n* = 42). Among patients with a comorbid condition pre-index (*n* = 42), 14.3% had known LN that did not meet the exclusion criteria of severe active LN within 90 days pre-index (Table 1).

**Table 1:** Patient demographics and clinical characteristics (enrolled set, *N* = 59)

	Enrolled set (*N* = 59)
**Age, years, mean (SD)**	36.8 (10.2)
**Female, n (%)**	53 (89.8)
**Caucasian/White, n (%)**	59 (100.0)
**Time since SLE diagnosis, years, n (%)**	
<1	9 (15.3)
1–5	18 (30.5)
6–10	18 (30.5)
11–15	6 (10.2)
>15	8 (13.6)
**SLE disease severity at index, n (%)**^**a**^	
Mild	10 (16.9)
Moderate	38 (64.4)
Severe	11 (18.6)
**SLE disease activity at index, n (%)**^**a**^	
Persistent	39 (66.1)
Flare	19 (32.2)
Remission	1 (1.7)
**Previously diagnosed with LN, n (%)**^**b**^	6 (14.3)
**SLE disease characteristics at index, n (%)**^**c**^	
Any of below	56 (94.9)
Low C3 (<LLN)	22 (37.3)
Low C4 (<LLN)	19 (32.2)
High anti-dsDNA	45 (76.3)
Proteinuria (>ULN)	2 (3.4)
Leukopenia	22 (37.3)
Thrombocytopenia	8 (13.6)
Hemolytic anemia	7 (11.9)
**Comorbid condition present pre-index reported in ≥10% patients with a comorbid condition, n (%)**^**d**^	
Any comorbid condition	42 (71.2)
Sjogren’s syndrome	12 (28.6)
Antiphospholipid syndrome	11 (26.2)
Hypertension	9 (21.4)
Cerebrovascular disease	7 (16.7)
Osteoporosis	6 (14.3)
Thyroid disease	6 (14.3)
**Comorbid conditions per patient pre-index, mean (SD)**	1.7 (1.2)
**SLE clinical manifestations per patient at/prior to index, mean (SD)**^**e**^	3.7 (1.7)
**SLE clinical manifestations reported in ≥10% of patients, n (%)**	
Immunologic	47 (79.7)
Increased anti-dsDNA levels	44 (74.6)
Low complement (C3, C4, or CH50)	25 (42.4)
Mucocutaneous	36 (61.0)
Rash	29 (49.2)
Alopecia	11 (18.6)
Hematologic	26 (44.1)
Leukopenia	18 (30.5)
Thrombocytopenia	7 (11.9)
Hemolytic anemia	6 (10.2)
Musculoskeletal	24 (40.7)
Arthritis	24 (40.7)
Central nervous system	13 (22.0)
Headache	11 (18.6)
Constitutional	10 (16.9)
Fatigue	6 (10.2)
Inability to taper glucocorticoids	10 (16.9)

*dsDNA* double-stranded deoxyribonucleic acid, *LLN* lower limit of normal, *SD* standard deviation, *SLE* systemic lupus erythematosus, *ULN* upper limit of normal

^a^SLE disease severity and activity were assessed based on physician judgement

^b^*N* = 42

^c^The denominator for individual conditions equals the patient count within “Any of below”

^d^The denominator for individual conditions equals the patient count within “Any comorbid condition”

^e^Clinical manifestations were based on physician assessment, and documented at index or up to 30 days prior to index

At or 30 days prior to index, the mean (SD) number of SLE clinical manifestations was 3.7 (1.7). The most reported clinical manifestation categories included immunologic (79.7%), mucocutaneous (61.0%), hematologic (44.1%), musculoskeletal (40.7%), central nervous system (22.0%), constitutional (16.9%), and inability to taper glucocorticoids (16.9%). The most frequently reported manifestations in the above categories at or prior to index included increased anti-dsDNA antibody (74.6%), rash (49.2%), low complement (C3, C4, or CH50; 42.4%), arthritis (40.7%), leukopenia (30.5%), headache (18.6%), and alopecia (18.6%; Table 1).

Most patients enrolled initiated IV belimumab in 2019 or 2021 (25.4%, *n* = 15 each); 15.3% (*n* = 9) of patients started IV belimumab within the first year of SLE diagnosis. The most common reasons (multiple reasons permitted) for initiating IV belimumab included the previous regimen not being effective and to decrease use of glucocorticoids (76.3%, *n* = 45 each; Table 2).

**Table 2:** Reasons for initiation and discontinuation of IV belimumab (enrolled set, *N* = 59)

	Enrolled set (*N* = 59)
**Reasons for initiation, n (%)**^**a**^	
Patient condition worsening	31 (52.5)
Previous regimen not effective	45 (76.3)
Previous regimen not well tolerated	15 (25.4)
Decrease use of glucocorticoids	45 (76.3)
Previous regimen inconvenient	5 (8.5)
Drug–drug interaction with previous medications	1 (1.7)
Other^b^	1 (1.7)
**Reasons for discontinuation, n (%)**^**a**^	
Patient request	1 (12.5)
Loss of insurance or reimbursement	3 (37.5)
Lost to follow-up	4 (50.0)
COVID-19 pandemic	1 (12.5)
Adverse reaction^c^	1 (12.5)
Patient moved away	1 (12.5)

*IV* intravenous

^a^More than one may apply

^b^Presence of clinically cured infiltrative tuberculosis; not possible to use immunosuppressants

^c^Recurrent ligature fistula of a postoperative scar of the Achilles tendon

Overall, for 59.3% of patients (*n* = 35/59), the physician who enrolled the patient was the same physician who initiated their IV belimumab therapy. The mean (SD) starting dose for IV belimumab was 9.7 (1.1) mg/kg. Most patients had no changes to their IV belimumab therapy during the first 6 months (78.0%, *n* = 46/59); among the remaining 22.0% of patients who had changes, 10.2% (*n* = 6/59) had a dose change (mean [SD] dose increase of 0.4 [1.5] mg/kg) and 13.6% (*n* = 8/59) discontinued IV belimumab (multiple reasons permitted).

Among patients who discontinued IV belimumab in the first 6 months of therapy, median (minimum, maximum) time to discontinuation for the seven patients with available data was 27.0 (14.0, 153.0) days. The most common reasons (multiple reasons permitted) were lost to follow-up (50.0%) and/or loss of insurance or reimbursement (37.5%; Table 2).

During the first 6 months of IV belimumab therapy, 10.2% (*n* = 6) of patients received all planned 8 doses and 45.8% (*n* = 27) received 6 doses. Overall, 89.8% (*n* = 53/59) of patients completed ≥6 months of IV belimumab therapy, 40.7% (*n* = 24/59) completed ≥24 months of treatment, and 83.3% (*n* = 20/24) were still receiving treatment at the time of data collection.

### Physician-assessed clinical improvements after 6 months

**Fig. 2:**
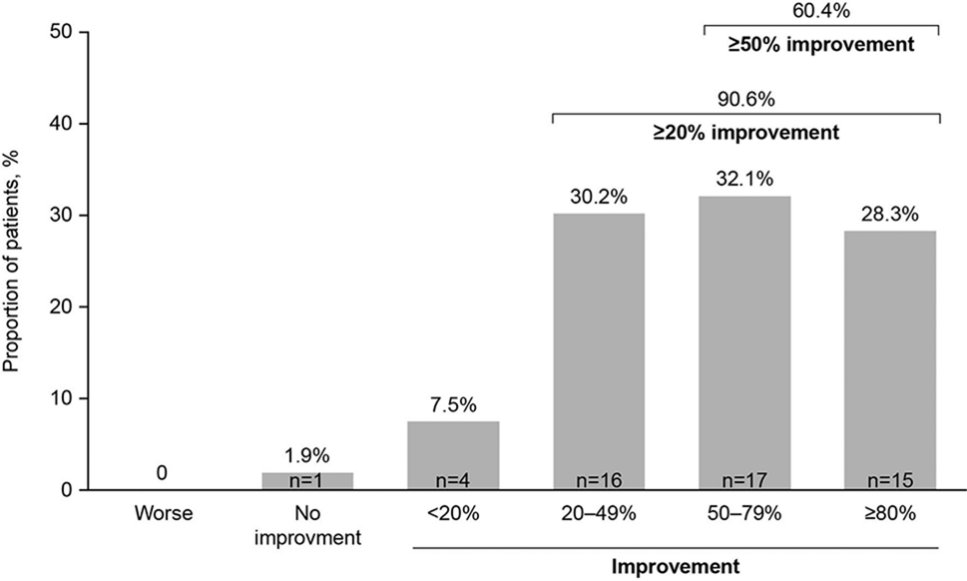
Physician-assessed overall clinical response to 6 months of IV belimumab treatment versus index date (FAS, *N* = 53). *FAS* full analysis set, *IV* intravenous

After 6 months of IV belimumab therapy, among the 53 patients who completed the treatment (FAS), 90.6% demonstrated an overall clinical improvement of ≥20%, while 60.4% exhibited an improvement of ≥50% (Fig. 2). Notably, no patient experienced a worsening of overall clinical response during the 6-month IV belimumab treatment.

### SLE disease activity assessment

A decrease in SLE disease activity was observed during the follow-up period. Among the enrolled population (*N* = 59), SELENA-SLEDAI was the most commonly used disease activity assessment tool, with score data available for 56 patients at index and 49 patients at 6 months post-index. The total mean (SD) SELENA-SLEDAI score decreased from 9.4 (3.7) at index to 3.7 (2.6) at 6 months post-index. Among the 48 patients with a score at both time points, the mean (SD) change in SELENA-SLEDAI score was −5.9 (4.3).

PhGA scores were documented for eight patients at both index and 6 months post-index. Among these patients, mean (SD) PhGA score decreased from 2.3 (0.5) at index to 1.0 (0.5) at 6 months post-index. Among the eight patients with data at both time points, the mean (SD) change from index in PhGA score was −1.3 (0.6).

SDI scores were documented for 11 patients at index and 8 patients at 6 months post-index. No change from index to 6 months post-index in SDI score was observed; mean (SD) SDI score was 1.0 (1.0) at index and 1.0 (1.1) at 6 months post-index.

### Tender and swollen joints

In the group of 24 patients with documented arthritis pre-index, improvements in joint health were evident. The overall mean (SD) tender joint count decreased from 5.1 (2.8) pre-index (*n* = 15) to 0.6 (1.3) post-index (*n* = 20). Similarly, the overall mean (SD) swollen joint count decreased from 2.9 (2.9) pre-index (*n* = 14) to 0.3 (0.4) post-index (*n* = 24), demonstrating positive outcomes in joint-related assessments.

### SLE flares

Of the 59 enrolled patients, flare data were available for 43 patients pre-index and 31 patients post-index. The proportion of patients experiencing ≥1 flare reduced from 79.1% (*n* = 34/43) pre-index to 22.6% (*n* = 7/31) post-index. No patient had more than two flares during the follow-up period; notable decline was observed in both mild/moderate and severe flare occurrences. The proportion of patients with ≥1 mild/moderate flare decreased from 69.8% (*n* = 30/43) pre-index to 19.4% (*n* = 6/31) post-index, and the proportion of patients with ≥1 severe flare decreased from 23.3% (*n* = 10/43) pre-index to 3.2% (*n* = 1/31) post-index.

### Concomitant SLE medications

Among patients who completed ≥6 months of IV belimumab therapy (FAS, *N* = 53), all patients received glucocorticoids at some point during the 12-month observational period; 94.3% (*n* = 50) of patients were receiving glucocorticoids both at index and at 6 months post-index (Table 3). The mean (SD) glucocorticoid dose decreased from 12.2 (7.3) mg/day at index to 8.6 (5.1) mg/day at 6 months post-index. Among the 49 patients with data at both time points, the mean (SD) change was −3.6 (5.2) mg/day. In this subgroup, 77.6% (*n* = 38) of patients were receiving ≥7.5 mg/day glucocorticoid dose at index; by 6 months post-index, eight of these patients decreased their dose to <7.5 mg/day and one discontinued glucocorticoid treatment completely. Among patients receiving <7.5 mg/day glucocorticoid dose at index (*n* = 11), only one patient shifted to ≥7.5 mg/day at 6 months post-index, with 10 patients continuing on <7.5 mg/day dose (Table 3).

**Table 3:** Summary of glucocorticoid use at index and during 6 months post-index

	FAS (*N* = 53)	
	Index	Post-index
**Glucocorticoid use, n (%)**	50 (94.3)	50 (94.3)
Discontinued	–	1 (2.0)
**Glucocorticoid dose, mg/day, mean (SD)**	12.2 (7.3)	8.6 (5.1)
Change from index to 6 months post-index^a^		–3.6 (5.2)
**Glucocorticoid dose category, n (%)**		
≥7.5 mg/day	38 (77.6)^a^	30 (62.50)^b^
<7.5 mg/day	11 (22.4)^a^	18 (37.5)^b^
**Change in dose category from index to 6 months post-index, n (%)**^**a**^		
Remained on <7.5 mg/day	–	10 (20.4)
Shifted from <7.5 mg/day to ≥7.5 mg/day	–	1 (2.0)
Shifted from ≥7.5 mg/day to discontinued	–	1 (2.0)
Remained on ≥7.5 mg/day	–	29 (59.2)
Shifted from ≥7.5 mg/day to <7.5 mg/day	–	8 (16.3)

*FAS* full analysis set, *SD* standard deviation

^a^Data for 49 patients with glucocorticoid dose data at both time points

^b^Data for 48 patients, excluding one patient who discontinued

Other concomitant SLE medications, excluding glucocorticoids, were analyzed in the enrolled set (*N* = 59) and were received by 91.5% of patients at index or during the 6 months pre-index (Table 4). The most frequently reported were antimalarials (96.3%), mycophenolate mofetil (22.2%), azathioprine (22.2%), and rituximab (13.0%). Disease-modifying antirheumatic drugs such as methotrexate, nonsteroidal anti-inflammatory drugs (NSAIDs), and cyclophosphamide were all reported for less than 10% of patients.

**Table 4:** Summary of concomitant SLE medication use (excluding glucocorticoids) received during the 6 months before index, or at index, and during 6 months post-index

Concomitant SLE medications, n (%)^a^	At or 6 months prior to index Enrolled set (*N* = 59)	During 6 months post-index Enrolled set (*N* = 59)
**Any of the below**	54 (91.5)	48 (81.4)
Antimalarials	52 (96.3)	45 (93.8)
Azathioprine	12 (22.2)	9 (18.8)
Mycophenolate mofetil	12 (22.2)	7 (14.6)
Rituximab	7 (13.0)	1 (2.1)
Methotrexate	5 (9.3)	6 (12.5)
Cyclophosphamide	1 (1.9)	-^b^
NSAIDs	1 (1.9)	-^b^

*NSAIDs* nonsteroidal anti-inflammatory drugs, *SLE* systemic lupus erythematosus

^a^More than one medication per patient allowed

^b^Data not documented

During 6 months post-index, the overall number of patients receiving concomitant medications (excluding glucocorticoids) reduced to 81.4% (Table 4). During the 6 months post-index, the proportions of patients receiving different medications were similar when compared with those receiving medications at index or during the 6 months pre-index, except for a notable decrease in those receiving mycophenolate mofetil (14.6%) and rituximab (2.1%).

### Laboratory results

Among the enrolled set (*N* = 59), 43 and 34 patients had anti-dsDNA data documented at index and 6 months post-index, respectively. Among these patients, the mean (SD) anti-dsDNA levels decreased from 124.8 (95.5) IU/ml at index to 87.6 (99.5) IU/ml at 6 months post-index.

Data for C3/C4 levels were documented for 30 and 25 patients at index and 6 months post-index, respectively. Among these patients, a small increase in the mean (SD) C3 levels was observed from 0.8 (0.2) g/l at index to 0.9 (0.2) g/l at 6 months post-index. No change was observed in the C4 levels; the mean (SD) C4 level was 0.1 (0.1) g/l at both index and 6 months post-index.

### HCRU

Among the FAS population (*N* = 53), 51 and 52 patients had recorded data on scheduled visits during pre- and post-index periods, respectively. Among these patients, the mean (SD) number of scheduled physician office visits during the pre-index period was 1.3 (1.0), increasing to 4.4 (2.8) during the 6 months post-index. The proportions of patients with ≥1 scheduled visit increased from 78.4% (*n* = 40) pre-index to 82.7% (*n* = 43) post-index (Table 5).

**Table 5:** Summary of HCRU in the 6 months pre- and post-index

Healthcare resources	During 6 months pre-index FAS (*N* = 53)	During 6 months post-index FAS (*N* = 53)
**Scheduled physician office visits**^**a**^	**51**	**52**
0 visits, n (%)	11 (21.6)	9 (17.3)
1 visit, n (%)	25 (49.0)	4 (7.7)
≥2 visits, n (%)	15 (29.4)	39 (75.0)
Number of visits/patient, mean (SD)	1.3 (1.0)	4.4 (2.8)
**Unscheduled physician office visits**^**a**^	**41**	**40**
0 visits, n (%)	30 (73.2)	38 (95.0)
1 visit, n (%)	9 (22.0)	1 (2.5)
≥2 visits, n (%)	2 (4.9)	1 (2.5)
Number of visits/patient, mean (SD)	0.3 (0.7)	0.2 (1.0)
**Hospitalizations**^**a**^	**26**	**25**
0 hospitalizations, n (%)	0 (0.0)	14 (50.0)
1 hospitalization, n (%)	19 (67.9)	3 (10.7)
≥2 hospitalizations, n (%)	7 (25.0)	8 (28.6)
Length of stay per hospitalization, nights, mean (SD)	12.3 (4.6)	3.6 (4.8)
**≥1 emergency room visit, n (%)**	2 (3.8)	2 (3.8)

*FAS* full analysis set, *HCRU* healthcare resource utilization, *SD* standard deviation

^a^Among patients with available data

Among patients with available data on unscheduled visits during pre- and post-index periods (41 and 40 patients, respectively), 26.8% (*n* = 11/41) during pre-index and 5.0% (*n* = 2/40) during post-index had ≥1 unscheduled physician office visit. The proportion of patients with no unscheduled physician office visits increased from 73.2% (*n* = 30) pre-index to 95.0% (*n* = 38) post-index (Table 5).

Overall, 26 patients in the pre-index period and 25 in the post-index period had data documented for hospitalizations, with 100% (*n* = 26) of these patients having ≥1 pre-index hospitalization and 44.0% (*n* = 11) having ≥1 post-index hospitalization (Table 5).

The mean (SD) number of nights in hospital decreased from 12.3 (4.6) pre-index to 3.6 (4.8) post-index.

Only two patients had documented information on emergency room visits; however, the number of emergency room visits and the period when they occurred were not reported (Table 5).

## Discussion

This retrospective, observational OBSErve study conducted in the Russian Federation aimed to describe the physician-assessed overall clinical response to IV belimumab and treatment patterns in adult patients with SLE in real-world settings. The results of this study demonstrated an early and sustained clinical response to IV belimumab during the first 6 months of treatment as part of standard SLE therapy. Notably, a majority of patients achieved an overall clinical improvement in disease activity, and overall reductions in SELENA-SLEDAI and PhGA scores were observed following belimumab treatment, providing insights into the positive impact of IV belimumab on disease activity. Moreover, the study highlights a marked decrease in the proportion of patients experiencing flares with belimumab treatment. The reduction in both mild/moderate and severe flares, despite the increase in scheduled visits post-index, suggests the potential of IV belimumab in preventing disease exacerbations, contributing to improved long-term outcomes. Joint health improvements were evident, with a notable reduction in both tender and swollen joint counts among patients with documented arthritis pre-index, suggesting the positive impact of IV belimumab on joint-related symptoms. No change in the SDI scores during the follow-up period indicates no new damage, suggesting that IV belimumab may contribute to prevention of long-term organ damage progression. Our results also demonstrated a decrease in glucocorticoid use, suggesting that IV belimumab may contribute to a dose-sparing effect. An overall reduction in HCRU was also observed.

IV belimumab contributed to early and sustained clinical responses in this patient population with a wide SLE duration ranging from <1 to >15 years. This suggests that IV belimumab could provide clinical benefit to patients with long-established SLE, although subgroup analyses by disease duration would be required to evaluate IV belimumab in such patients. Overall, these findings are in agreement with those reported in previous randomized clinical trials for IV belimumab, and in real-world IV belimumab studies in several countries and populations [11–15, 18].

In this OBSErve Russia study, IV belimumab was generally initiated to help patients achieve the desired disease control or to reduce their glucocorticoid intake/usage. In this study, the overall clinical response appears to be higher than in other OBSErve studies included in a pooled analysis by Collins et al., with 90.6% versus 82.8% of patients demonstrating an overall clinical improvement of ≥20% and 60.4% versus 48.1% demonstrating an improvement of ≥50% after 6 months of IV belimumab treatment [18].

Minimizing glucocorticoid use is an important goal in the management of SLE [4]. In OBSErve Russia, over 90% of patients were receiving glucocorticoids at IV belimumab initiation. While only one patient was able to discontinue glucocorticoids after 6 months of IV belimumab treatment, nearly 40% remained on, or shifted to a low dose (<7.5 mg/day) and, overall, patients experienced a mean reduction in glucocorticoid dose of 3.6 mg/day. This steroid-sparing effect of IV belimumab treatment has previously been observed in IV belimumab trials [19, 20] and is consistent with that seen in other OBSErve studies and their pooled analysis [10–12, 14, 15, 18].

In this study, we also evaluated the extent of HCRU in patients with SLE in the Russian Federation receiving IV belimumab. As expected, the number of scheduled physician office visits increased during the course of IV belimumab therapy, which requires monthly infusions at clinic. It is possible that the increased visits meant that patients were monitored more closely after IV belimumab initiation than before, which could have led to an increased perception of clinical response by the treating physician. However, the reduction in the number of patients with unscheduled visits and hospitalizations post-index, combined with the decrease in hospitalization durations with IV belimumab treatment, point to improvements in SLE disease management with 6 months of IV belimumab treatment, independently of the physicians’ perceptions of clinical response.

Over half of the patients were initiated on IV belimumab by their current treating physician. The mean IV belimumab starting dose (9.7 mg/kg) was slightly lower than the recommended 10 mg/kg IV dosing; this can be explained by the availability of two sizes of IV belimumab packs (120 mg and 400 mg) in the Russian Federation. To limit wastage and use the available vials fully, in routine clinical practice IV belimumab dose is recalculated with rounding the patient’s weight to within 1–2 kg. Only a minor proportion of patients required a dose change (10.2%) or discontinued IV belimumab (13.6%) during the first 6 months of therapy. The discontinuation of IV belimumab therapy was mainly due to a loss to follow-up and/or loss of insurance or reimbursement. A considerable proportion of patients (89.9%) did not receive the standard therapy dosage of eight IV belimumab infusions over the 6-month follow-up period, perhaps due to an existing algorithm of allocation of quotas for treatment and patients’ individual circumstances (e.g., logistics around travel to clinic) in different regions in the Russian Federation. Ultimately, approximately 83% of patients were still receiving IV belimumab at the time of data collection. These results indicate a well-established relationship with prescribing physicians, good adherence to IV belimumab treatment, and a high retention rate.

Observational studies such as this are more reflective of clinical practice than clinical studies, which have strict criteria and protocols; however, there are limitations to consider, including the lack of a control group, the descriptive nature of the study, and the small number of patients. In addition, patients and investigation procedures may have varied across different sites and may not be representative of non-participating sites. The inclusion criterion for an SLE diagnosis without the requirement to fulfill a recognized classification could have resulted in misclassification in some patients in the absence of an objective, differential diagnosis recorded by the treating physician. The primary endpoint of clinical response was evaluated using a physician-deemed clinical improvement scale, which is inherently dependent on the individual, subjective clinical judgement of the treating physician, following retrospective review of medical records, and this represents a notable limitation. Furthermore, despite recruitment of consecutive patients, the requirement of ≥6 months belimumab treatment among the primary analysis cohort may introduce a risk of recruitment bias towards patients who had a beneficial response to IV belimumab; exhibited favorable tolerance to IV belimumab with limited safety events; were compliant with IV belimumab and patient visits; or were treated by reporting physicians who were compliant with regular patient follow-up. Also, despite physicians being asked to enroll patients consecutively, starting with the most recent patient to have completed 6 months of IV belimumab treatment and working backwards, the identification of patients by physicians may have differed between sites, consisting of a mix of both paper and electronic medical records, which could introduce a sampling bias and impact the generalizability of the results. Nevertheless, the real-world setting of this study enhances its external validity, providing insights into the practical effectiveness of IV belimumab in a diverse and authentic clinical environment. The multifaceted evaluation of SLE disease activity, which consistently showed improvement across all measured parameters, strengthens the study’s findings, further reinforcing the efficacy of belimumab demonstrated in previous clinical trials. This study provides important additional validation of the effectiveness of IV belimumab in patients with SLE in a real-world setting and may provide evidence for appropriate treatment regimens in clinical practice.

## Conclusions

In this retrospective, observational OBSErve study conducted in the Russian Federation, the majority of patients receiving IV belimumab for 6 months achieved clinically meaningful improvements in their SLE disease activity, as measured by the physician’s assessment as well as SELENA-SLEDAI and PhGA assessment. Improvements in overall SLE disease activity allowed for reduction or discontinuation of glucocorticoids in some patients as well as the reduction in HCRU. These findings support the results of clinical trials of IV belimumab and other real-world evidence studies.

## Electronic supplementary material

Below is the link to the electronic supplementary material.

Additional File 1 Systemic lupus erythematosus (SLE) flare definition

## Data Availability

All data generated and analyzed during this study are included in the manuscript and its supplementary information file.

## Abbreviations

ACR: American College of Rheumatology
BLyS: B-lymphocyte stimulator
CI: Confidence interval
eCRFs: Electronic case report forms
FAS: Full analysis set
HCRU: Healthcare resource utilization
IgG1λ: Immunoglobulin G1λ
IV: Intravenous
LN: Lupus nephritis
NSAIDs: Nonsteroidal anti-inflammatory drugs
PhGA: Physician Global Assessment
SC: Subcutaneous
SD: Standard deviation
SDI: SLICC/ACR Damage Index
SELENA-SLEDAI: Safety of Estrogens in Lupus Erythematosus National Assessment – Systemic Lupus Erythematosus Disease Activity Index
SLE: Systemic lupus erythematosus
SLICC: Systemic Lupus International Collaborating Clinics
