# Supplementary material for: Real-world effectiveness of intravenous belimumab in adults with systemic lupus erythematosus: results of the observational OBSErve study in the Russian Federation
Source: BMC Rheumatol. 2025 Jan 8;9:4. doi: 10.1186/s41927-024-00452-0 (PMC11708191; doi:10.1186/s41927-024-00452-0)
Supplement: Supplementary file 1 — Additional File 1 Systemic lupus erythematosus (SLE) flare definition [file 41927_2024_452_MOESM1_ESM.pdf]

Additional file 1

Systemic lupus erythematosus (SLE) flare definition

The mild/moderate flare definition was based on the Safety of Estrogens in Lupus National Assessment-SLE Disease Activity Index (SELENA-SLEDAI) flare composite,[1,2] and was defined as one or more of the following:

- Change in SELENA-SLEDAI score by ≥3 points (with a total score of 12 or less)
- New/worsening:
  - Discoid, photosensitive, or other rash attributable to lupus (including lupus profundus, cutaneous vasculitis, or bullous lupus)
  - Nasopharyngeal ulcers
  - Pleuritis
  - Pericarditis
  - Arthritis
  - Fever not attributable to infection
- Increase in daily prednisone-equivalent dose but not greater than 0.5 mg/kg
- Initiation of therapy with either hydroxychloroquine or nonsteroidal anti-inflammatory drugs (NSAIDs) without an increase in prednisone dose
- Change in the Physician Global Assessment (PhGA) score by ≥1 but remaining ≤2.5

The severe flare definition was based on the SELENA-SLEDAI flare composite,[1,2] and was defined as one or more of the following:

- SELENA-SLEDAI score >12
- New/worsening:
  - Central nervous system involvement
  - Vasculitis
  - Glomerulonephritis
  - Myositis
  - Thrombocytopenia (platelet count <60 x 10^9 cells/l)
  - Hemolytic anemia (hemoglobin level <70 g/l or decrease in hemoglobin level >30 g/l over a 2-week period), requiring doubling of daily glucocorticoid dose to a final >0.5 mg/kg or acute hospitalization
- Any manifestation requiring an increase in daily prednisone-equivalent dose to >0.5 mg/kg

- Initiation of therapy with cyclophosphamide, azathioprine, mycophenolate mofetil, or methotrexate
- Hospitalization for SLE activity
- Change in PhGA score from baseline to >2.5

References

1. Petri M, Kim MY, Kalunian KC, Grossman J, Hahn, BH, Sammaritano, LR, et al. Combined oral contraceptives in women with systemic lupus erythematosus. N Engl J Med. 2005;353:2550-8.

2. Petri M, Buyon J, Kim M. Classification and definition of major flares in SLE clinical trials. Lupus. 1999;8(8):685-91.
